# Factors associated with smoking intensity among adult smokers: findings from the longitudinal cohort of the Tehran lipid and glucose study

**DOI:** 10.1186/s12889-023-17232-z

**Published:** 2023-12-15

**Authors:** Marjan Abbasi-Dokht-Rafsanjani, Samaneh Hosseinzadeh, Enayatollah Bakhshi, Fereidoun Azizi, Davood Khalili

**Affiliations:** 1 Department of Biostatistics and Epidemiology, University of Social Welfare and Rehabilitation Sciences, Tehran, Iran; 2 Endocrine Research Center, Research Institute for Endocrine Sciences, Shahid Beheshti University of Medical Sciences, Tehran, Iran; 3 Prevention of Metabolic Disorders Research Center, Research Institute for Endocrine Sciences, Shahid Beheshti University of Medical Sciences, Tehran, Iran; 4 Division of Biostatistics and Epidemiology, Research Institute for Endocrine Sciences, Shahid Beheshti University of Medical Sciences, Tehran, Iran

**Keywords:** Smoking, Smoking intensity, Cigarette, Adults, TLGS

## Abstract

**Background:**

Smoking is a significant public health problem, and there is a scarcity of documents regarding its severity, particularly in developing countries. This study aimed to determine factors related to the number of cigarettes consumed daily by adult smokers in Tehran.

**Methods:**

This study was conducted within the framework of the longitudinal study of Tehran Lipid and Glucose Study (TLGS). The study included 786 adult smokers living during four consecutive follow-ups from 2005 to 2016. The intensity of smoking was measured by the number of cigarettes consumed daily by adult smokers. Data analysis was done longitudinally and based on the mixed effects zero-inflated discrete Weibull (ZIDW) regression model.

**Results:**

The mean age of the individuals was 40.35 ± 12.68 years, and 643 (81.8%) of them were men. Also, 52.7% of individuals were daily smokers, 15.6% were occasional smokers, and 31.7% were non-smokers who became smokers during the study. Variables of age 1.005 (95%CI: 1.001–1.008), gender of male 1.196 (95%CI: 1.051–1.39), and marital status (divorced/widowed vs. single) 1.168 (95%CI: 1.015–1.39) were positively associated with smoking intensity. Education level (master and higher vs. illiterate) 0.675 (95%CI: 0.492–0.926)), employment status (student vs. unemployed) 0.683 (95%CI: 0.522–0.917), (housewife vs. unemployed) 0.742 (95%CI: 0.606–0.895), (Unemployed with income vs. unemployed) 0.804 (95%CI: 0.697, 0.923), implementation of smoking prohibition regulations (yes vs. no) 0.88 (95%CI: 0.843–0.932), and history of cardiovascular disease in male relatives (yes vs. no) 0.85 (95%CI: 0.771–0.951) were associated with lower smoking intensity.

**Conclusion:**

We showed that demographic factors are associated with the intensity of smoking among adults and should be considered in policymakers’ intervention programs to reduce smoking and quit smoking.

## Introduction

 Smoking is a major public health concern worldwide and is the leading cause of preventable death and disease. The World Health Organization reports that in 2020, 22.3% of the world’s population consumed tobacco [1]. In Iran, the prevalence of smoking among adults has increased over the years, with men being more affected than women [2]. Smoking is responsible for 7 million deaths annually, and if the trend continues, it is estimated that 8 million people will die annually from tobacco-related diseases by 2030 [3]. Smoking is a risk factor for various diseases, including cardiovascular diseases, asthma, pneumonia, lung cancer, and other cancers [4]. Smokers are at a higher risk of dying from respiratory and heart diseases than non-smokers [5].

Smoking is the leading preventable cause of death worldwide, and it is crucial to identify the factors that influence its use [6]. The pattern of smoking is influenced by important factors such as individual and demographic, social and economic factors. While many studies have been conducted to investigate the prevalence of smoking and the factors related to it worldwide, there is still a lack of research aimed at identifying the factors related to the intensity of smoking (number of cigarettes smoked per day) in the adult smoking population.

The number of cigarettes smoked daily and the duration of smoking are critical factors that contribute to the risk of developing smoking-related diseases and deaths [7]. Even smoking one cigarette a day can increase heart rate and blood pressure [8]. Additionally, Each cigarette smoked cuts someone’s life by 11 min on average; hence, quitting smoking is the most important change that smokers can make to improve their health [9]. Smoking more cigarettes per day is associated with serious adverse consequences for one’s health, as well as social and economic issues. Count regression models can be used to determine factors related to smoking intensity. Reducing cigarette consumption can improve individual health and increase household budgets for education, health, and better livelihood, even if quitting smoking altogether seems unattainable.

This study aims to identify factors related to smoking intensity among adult smokers in Tehran using the Bayesian mixed effects zero-inflated discrete Weibull (ZIDW) regression model. Identifying factors affecting smoking intensity is crucial for planning and implementing appropriate interventions to change this risky behavior and reduce the burden of tobacco-related.

## Methods

### Participants and design

The current longitudinal cohort study has been conducted within the framework of the Tehran Lipid and Glucose Study (TLGS). The TLGS is an ongoing population-based longitudinal cohort study that investigates the prevalence and incidence of non-communicable diseases and their risk factors in over 15,000 individuals aged ≥ 3 years residing in district 13 of Tehran. The design of the TLGS included two main parts: First phase, a cross-sectional prevalence study of non-communicable diseases and their risk factors (1999–2001); and prospective ongoing follow-up examinations which have been continued every 3 years. Details of the rationale, sampling and data collection of the TLGS have been reported elsewhere [10, 11].

This longitudinal analysis was conducted on data the third (2005–2007) to sixth (2014–2016) phases of the TLGS. The study analyzed data from adults (over 18 years old) who participated in the third to sixth phases of the TLGS and reported smoking daily or occasionally in at least one of these phases. Initially, we selected adults who smoked daily or occasionally in at least one of the four phases. From a total of 2,107 adult participants, 1,210 were excluded because they were lost to follow-up. Of the remaining 897 adults who participated in all four phases and reported smoking daily or occasionally in at least one phase, 111 were excluded due to incomplete data (missing demographic information or smoking questionnaire). Finally, we included 786 adult smokers (daily or occasional) who participated in all four phases of the TLGS study and had complete data for analysis.

### Data collection

Demographic information, including age, sex, marital status, employment status, and level of education, as well as medical records questionnaire of individuals, including history of premature cardiovascular disease (CVD) in male and female relatives, were collected through individual interviews by trained personnel. Participants were also asked about their smoking behavior, including the number of cigarettes consumed daily, the number of days smoked per week, and the status of implementation of smoking prohibition regulations at work or school.

The research ethics committee of university of Social Welfare Rehabilitation Sciences approved this study (IR.USWR.REC.1400.298). In the TLGS study, informed written consent was obtained from all participants.

### Outcome measurement

Individuals were categorized as non-smokers (not using any cigarettes or tobacco in the past or present), active or daily smokers (using at least 7 cigarettes per week or an average of at least one cigarette per day), or inactive or occasional smokers (less than 7 cigarettes per week) [12]. The TLGS study collected information on smoking status as well as the number of cigarettes smoked per day by adult smokers. In the adult smoking questionnaire of the TLGS study, participants were first asked whether they smoke (daily or occasionally) or not. For respondents who smoke (daily or occasionally), “yes” was selected, otherwise “no”. For respondents who reported smoking, a follow-up question asked about the number of days per week they smoked. Occasional smokers (smoking only some days of the week) smoke fewer days per week than daily smokers; for this reason, the answer of the participants to this question is of special importance. A subsequent question asked about the number of cigarettes smoked daily by participants. This study used the response to this question to identify the factors related to the smoking of intensity among adult smokers. The number of cigarettes smoked per day by each adult was considered as the outcome variable, which is a count response.

### Explanatory variables

Sex, marital status (single, married, divorced/ widowed), employment status (unemployed, employed, student, housewife, unemployed with income, other), level of education (secondary/diploma, Up to bachelor’s level, Master’s degree and higher), history of premature cardiovascular disease in male relatives (father, brother, or son), history of premature cardiovascular disease in female relatives (mother, sister, or daughter), status of implementation of regulations prohibiting smoking at work or school (no, yes and I am not a student or a Employed), and the number of days of smoking per week were considered in a qualitative-nominal form (except for the age variable, which is included continuously).

### Statistical analysis

Quantitative and qualitative data were described using mean (standard deviation) and frequency (percentage), respectively. The number of cigarettes consumed daily by each adult is a count response, and therefore, Count regression models can be used to determine factors related to smoking intensity. Recently has been proposed a Bayesian mixed effects ZIDW regression model for longitudinal count data as an alternative to mixed effects regression models that are based on the usual NB, ZINB, and conventional DW distribution; that this model for longitudinal count data provides better fits than its competitors when the data are skewed and contain excess zeros [13].

In the current study, excess zeros and overdispersion (the variance of the number of cigarettes smoked daily in each of the follow-up was larger than its mean) in the number of cigarettes smoked per day data exist. Furthermore, the mean cigarettes smoked count may greatly exceed the median count because the distribution of the data is skewed and overdispersed, so that the median count might be a more appropriate characteristic for the investigating factors affecting the intensity of smoking than the mean. Therefore, we used the mixed effects zero-inflated discrete Weibull (ZIDW) regression model with the Bayesian approach to specify the relationship between the linear predictors and the median counts using the log-link function. Data analysis was done using JAGS (Just Another Gibbs Sampler) version 4.3.0 and R software version 3.5.1. Also, in this study, the mixed effects zero inflated negative binomial (ZINB) regression model was fitted to compare with the Bayesian mixed effects zero inflated discrete Weibull (ZIDW) regression model and according to the comparison of the sum of the standard deviations of the parameters of the regression models, as well as the 95% highest posterior density (HPD) intervals, the Bayesian mixed effects zero inflated discrete Weibull (ZIDW) regression model was selected for data analysis due to its better fit.

### Zero inflated discrete Weibull (ZIDW) regression model

Due to the correlation between observations in longitudinal data, fixed effects models cannot be used because the main assumption of the fixed effects model is that the observations are independent. Generalized linear mixed models are a useful method for analyzing such data [14]. In statistical modeling, when the data distribution is highly skewed and overdispersed, the mean response count may greatly exceed the median count so that the median count might be a more appropriate characteristic for the evaluating the factors affecting the count than the mean [15]. The mixed effects ZIDW regression model uses the log-link function to specify the relationship between the linear predictors and the median counts, therefore offering a robust characteristic of central tendency, compared to the mean count, when the distribution of the data is skew [13].

Suppose that ${y}_{ij}$ is the count outcome for patient (i = 1,…,N) at time point j = 1,…,T_i_. Furthermore, β &${\text{u}}_{\text{i}}$are, respectively, vectors of fixed and patient-specific random effects, and ${\text{x}}_{\text{i}\text{j}}$&${ \text{z}}_{\text{i}\text{j}}$are covariate vectors, respectively, containing baseline characteristics and measurement times. Assume the ${\text{u}}_{\text{i}}$ follow multivariate normal distributions with mean 0 and d-dimensional unstructured covariance matrix Σ, such that u_i_ ∼ N_d_ (0,∑). The probability mass function of the ZIDW regression model for a given count ${y}_{ij}$ over time is written as: 

1 $$\text{f}\left({\text{y}}_{\text{i}\text{j}}\mid {\upbeta },{\text{u}}_{\text{i}},{\uprho },{\uppi }\right)={\uppi }\text{I}\left({\text{y}}_{\text{i}\text{j}}=0\right)+(1-{\uppi })\times \left[\text{e}\text{x}\text{p}\left(-\text{l}\text{o}\text{g}\left(2\right){\left[\frac{{\text{y}}_{\text{i}\text{j}}}{{\text{e}}^{{\text{x}}_{\text{i}\text{j}}^{{\prime }}{\upbeta }+{\text{z}}_{\text{i}\text{j}}^{{\prime }}{\text{u}}_{\text{i}}}}\right]}^{{\uprho }}\right)-\text{e}\text{x}\text{p}\left(-\text{l}\text{o}\text{g}\left(2\right){\left[\frac{{\text{y}}_{\text{i}\text{j}}+1}{{\text{e}}^{{\text{x}}_{\text{i}\text{j}}^{{\prime }}{\upbeta }+{\text{z}}_{\text{i}\text{j}}^{{\prime }}{\text{u}}_{\text{i}}}}\right]}^{{\uprho }}\right)\right]$$

Here, 𝜌 and 𝜋 are, respectively, the shape parameter and zero inflation probability of the ZIDW distribution [16]. The median of the ${y}_{ij}$ under the ZIDW regression model is given by [13]:

2 $$\text{M}\left({\text{y}}_{\text{i}\text{j}}\right)+1={\left(\frac{\text{l}\text{o}\text{g}\left[\frac{0.5}{1-{\uppi }}\right]}{\text{l}\text{o}\text{g}\left(0.5\right)}\right)}^{1/{\uprho }}{\text{e}}^{{\text{x}}_{\text{i}\text{j}}^{{\prime }}{\upbeta }+{\text{z}}_{\text{i}\text{j}}^{{\prime }}{\text{u}}_{\text{i}}}$$

### Bayesian specification

For each component of β, a normal prior distribution, N (0,10000), is assigned. The dispersion and shape parameters are specified a gamma prior distribution, denoted as G (0.5,0.5). Also, the zero inflation probability is assigned a uniform prior distribution, denoted as U (0,1) [13]. The Wishart prior distribution is informative when the variances are close to zero [17], so in order to provide a more suitable alternative to the commonly used Wishart prior distribution, the MGH-t prior distribution is chosen for the variance-covariance (Σ) matrix [13]. The MGH-t prior distribution is expressed as a mixture representation of G(0.5,1/A^2^) for the diagonal entries of diagonal matrix ${\Omega }=\text{diag}\left({\omega }_{1},\dots ,{\omega }_{k},\dots ,{\omega }_{d}\right),$ and the Wishart distribution with inverse scale matrix 2vΩ and degrees of freedom $v+d-1$, namely, $W(2v{\Omega } ,v+d-1)$, with corresponding values A = 10,000 and v = 2 [18]. This mixture representation leads to determining the half-t prior distribution, i.e., half-t (v, A), for the standard deviations in Σ and the uniform prior distribution, i.e., U(-1,1), for the correlations in Σ. The probability density function of Σ is written as:

$$P\left({\Sigma }\right)\propto |{\Sigma }{|}^{-(v+2d)/2}\prod _{k=1}^{d} {\left[v{\left({{\Sigma }}^{-1}\right)}_{kk}+1/{A}^{2}\right]}^{-(v+d)/2}$$

Where ∑ > 0. Here, ${\left({{\Sigma }}^{-1}\right)}_{kk}$ denotes the kth diagonal entry of 𝚺^−1^.

Let ${\text{y}}_{\text{i}}$ denote T_i_×1 vectors containing $\left({y}_{i1},\dots ,{y}_{ij},\dots ,{y}_{i{T}_{i}}\right){\prime }$. The resulting joint posterior distribution of the model parameters can be written as:

$$P\left(\beta ,{u}_{i},\rho ,\pi ,{\Sigma },{\omega }_{k},i=1,\dots ,N,k=1,\dots ,d\mid y\right) \propto \left[{\prod }_{i=1}^{N} {\prod }_{j=1}^{{T}_{i}} f\left({y}_{ij}\mid \beta ,{u}_{i},\rho ,\pi \right)\right]P\left(\beta \right)\left[{\prod }_{i=1}^{N} P\left({u}_{i}\mid {\Sigma }\right)\right]P\left(\rho \right)P\left(\pi \right)P\left({{\Sigma }}^{-1}\mid {\Omega }\right)\left[{\prod }_{k=1}^{d} P\left({\omega }_{k}\right)\right],$$

Where y denotes the ${\sum }_{i=1}^{N} {T}_{i}\times 1$vector containing ${\text{y}}_{\text{i}}$ for all $\text{i}=1 ,\dots ,\text{N}.$ The corresponding probability density functions are written as [13]:

$$\begin{array}{rl}P\left(\beta \right)& \propto \text{e}\text{x}\text{p}\left(-0.00005{\beta }^{{\prime }}\beta \right)\\ P\left({u}_{i}\mid {\Sigma }\right)& \propto |{\Sigma }{|}^{-\frac{1}{2}} \text{e}\text{x}\text{p}\left(-\frac{1}{2}{u}_{i}^{{\prime }}{{\Sigma }}^{-1}{u}_{i}\right)\\ P\left(\rho \right)& \propto {\rho }^{-\frac{1}{2}} \text{e}\text{x}\text{p}\left(-\frac{1}{2}\rho \right)\\ P\left(\pi \right)& \propto 1\\ P\left({{\Sigma }}^{-1}\mid {\Omega }\right)& \propto \text{e}\text{x}\text{p}\left[-2\text{t}\text{r}\left({\Omega }{{\Sigma }}^{-1}\right)\right];{\Omega }=\text{d}\text{i}\text{a}\text{g}\left({\omega }_{1},\dots ,{\omega }_{k},\dots ,{\omega }_{d}\right)\\ P\left({\omega }_{k}\right)& \propto {\omega }_{k}^{-\frac{1}{2}}\text{exp}\left(-\frac{1}{{10}^{8}}{\omega }_{k}\right)\end{array}$$

The MCMC Gibbs sampling algorithm was used to draw samples from the joint posterior distribution of the model parameters [19]. The regression model was fitted using JAGS via the package runjags of the R project. Posterior samples were monitored and convergence was confirmed using iteration and autocorrelation plots, and Brooks-Gelman-Rubin statistics of parallel chains [13]. For regression model, 7,550,000 samples were simulated from the joint posterior distribution for three parallel chains. Among those 7,550,000 samples (per chain), the initial 50,000 samples were discarded (burn-in). The thinning factor was set to 50,000 to reduce autocorrelation among the samples [13].

## Results

The mean age of the participants at baseline was 40.35 ± 12.68 years, with a range of 19 to 75 years. Approximately half of the participants were between the ages of 30 and 49 years. The majority of the participants were male (81.8%), and about three-quarters were married (75.7%). Most participants had completed at least a diploma-level education (78.1%), and more than two-thirds were employed (68.4%). Baseline characteristics of the study population according smoking status are presented in Table 1.

**Table 1:** Sociodemographic characteristics of participants based on smoking status at baseline of the study (N = 786)

Variables	Daily smoker n(%)	Occasionally smoker n(%)		Non-smoker^a^ n(%)	Total n(%)	*P*-value
**Age**						
19–29	46(25.4)	30(16.6)	105(58.0)		181(23.0)	< 0.001
30–39	95(49.7)	30(15.7)	66(34.6)		191(24.3)	
40–49	146(61.9)	37(15.7)	53(22.5)		236(30.0)	
50–59	85(73.9)	13(11.3)	17(14.8)		115(14.6)	
60≤	42(66.7)	13(20.6)	8(12.7)		63(8.0)	
**Gender**						
Male	376(58.5)	96(14.9)	171(26.6)		643(81.8)	< 0.001
Female	38(26.6)	27(18.9)	78(54.5)		143(18.2)	
**Marital status**						
Single	51(30.2)	28(16.6)	90(53.3)		169(21.5)	< 0.001
Married	350(58.8)	91(15.3)	154(25.9)		595(75.7)	
Divorced/ Widowed	13(59.1)	4(18.2)	5(22.7)		22(2.8)	
**Employment status**						
Employed	307(57.1)	84(15.6)	147(27.3)		538(68.4)	< 0.001
Unemployed	18(46.2)	4(10.3)	17(43.6)		39(5.0)	
Student	2(7.7)	5(19.2)	19(73.1)		26(3.3)	
Housewife	25(27.2)	19(20.7)	48(52.2)		92(11.7)	
Unemployed with income	61(74.4)	10(12.2)	11(13.4)		82(10.4)	
Other	1(11.1)	1(11.1)	7(77.8)		9(1.1)	
**Education**						
Illiterate	8(80.0)	2(20.0)	0(0.0)		10(1.3)	0.063
Secondary/diploma	335(54.6)	94(15.3)	185(30.1)		614(78.1)	
Up to bachelor’s level	62(42.8)	25(17.2)	58(40.0)		145(18.4)	
Master’s degree and higher	9(52.9)	2(11.8)	6(35.3)		17(2.2)	
**Regulations prohibiting smoking at work or school**						
Yes	103(48.6)	32(15.1)	77(36.3)		212(27.0)	< 0.001
No	285(58.4)	80(16.4)	123(25.2)		488(62.1)	
I am not a student or a Employed	26(30.2)	11(12.8)	49(57.0)		86(10.9)	
**History of premature CVD in male relatives**						
No	362(52.7)	108(15.7)	217(31.6)		687(87.4)	0.984
Yes	52(52.5)	15(15.2)	32(32.3)		99(12.6)	
**History of premature CVD in female relatives**						
No	359(51.1)	112(15.9)	232(33.0)		703(89.4)	0.026
Yes	55(66.3)	11(13.3)	17(20.5)		83(10.6)	
**Number of smoking days per week**						
0	0(0.0)	0(0.0)	249(100)		249(31.7)	< 0.001
< 1	0(0.0)	46(100)	0(0.0)		46(9.5)	
1–2	0(0.0)	39(100)	0(0.0)		39(5.0)	
3–5	0(0.0)	35(100)	0(0.0)		35(4.5)	
6≤	414(99.3)	3(0.7)	0(0.0)		417(53.1)	

Note: The percentages were reported row-wise in columns 3, 4, and 5, and column-wise in column 6

^a^Non-smoker: These participants were non-smokers at baseline of the study and became smokers during the follow-ups

**Fig. 1:**
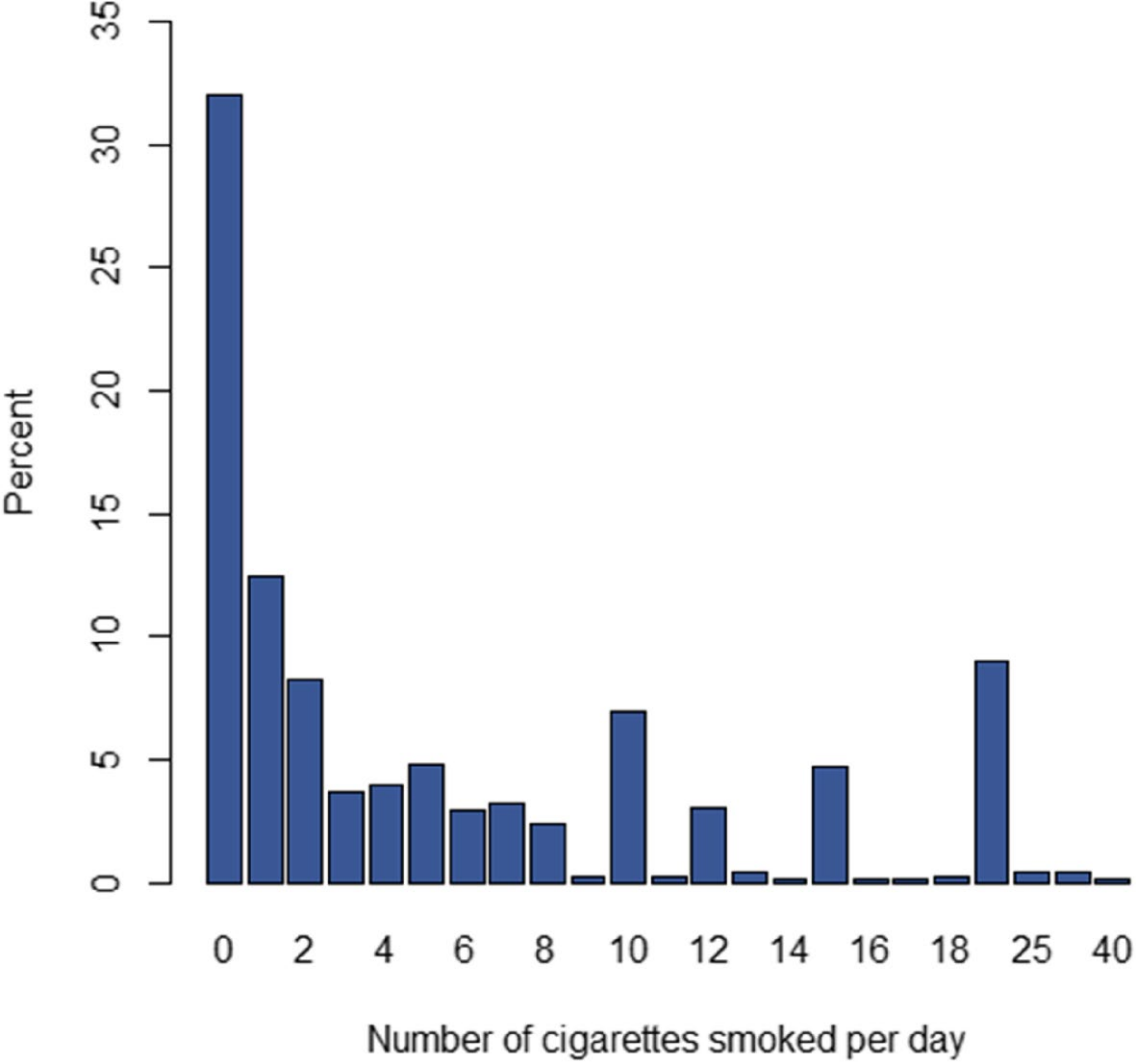
Frequency distribution of the number of cigarettes smoked daily among participants at baseline

At baseline, 414 participants (52.7%) were classified as daily smokers, 123 (15.6%) as occasional smokers, and 249 (31.7%) as non-smokers. Some participants who were non-smokers at baseline became smokers during study follow-ups. Figure 1 shows the frequency distribution of the number of cigarettes smoked daily by participants at baseline of the study (Phase 3 of the TLGS).

According to the Fig. 1, the data contains extra zeros (32%), indicating zero inflation. Additionally, the data distribution has a positive skewness. The ZIDW approach offers a more robust characteristic of central tendency, compared to the mean count, when there is skewness in the data [13].

Table 2 shows the results Bayesian mixed effects ZIDW regression model for the number of cigarettes smoked per day among adult smokers, which includes Exponentiated posterior estimates (PEs) and 95% highest posterior density (HPD) intervals of model parameters.

**Table 2:** Bayesian mixed effects ZIDW regression model for smoking intensity among adult smokers in Tehran

Variables (references)			β^a^		Exp(β)		95%CI^b^	
**Age**			0.005		1.005		1.001–1.008	
**Gender (female)**			0.179		1.196		1.051–1.39	
**Marital status (single)**								
Married			0.017		1.017		0.932–1.115	
Divorced/ Widowed			0.156		1.168		1.015–1.39	
**Education (illiterate)**								
secondary/diploma			-0.190		0.826		0.612–1.072	
Up to bachelor’s level			-0.243		0.784		0.588–1.042	
Master’s degree and higher			-0.393		0.675		0.492–0.926	
**Employment(Unemployed)**								
Employed			-0.111		0.894		0.794–1.015	
Student			-0.381		0.683		0.522–0.917	
housewife			-0.298		0.742		0.606–0.895	
unemployed with income			-0.218		0.804		0.697–0.923	
other			-0.105		0.90		0.576–1.419	
**History of premature CVD in male relatives (No)**			-0.162		0.85		0.771–0.951	
**History of premature CVD in female relatives (No)**			0.099		1.104		0.995–1.228	
**Regulations prohibiting smoking at work or school (No)**								
Yes			-0.127		0.88		0.843–0.932	
I am not a student or a Employed			-0.055		0.946		0.886–1.021	
**Number of smoking days per week (0)**								
< 1			2.567		13.026		8.248–22.421	
1–2			2.802		16.477		10.59-28.789	
3–5			3.121		22.669		14.731–40.447	
6≤			3.82		45.604		29.078–75.188	

Dependent variable: The number of cigarettes smoked per day (Smoking intensity) Note: The log-link function is specified to describe the relationship between the linear predictors and the median of the response variable. ^a^ *PE* Posterior estimate ^b^ 95% HPD intervals were reported for Exp (PE)

### Factors associated with smoking intensity

The analysis revealed that several demographic and lifestyle factors were significantly associated with the number of cigarettes consumed daily.

First, sex was found to have a significant effect on smoking intensity. The number of cigarettes smoked daily by male smokers is 1.196 times that of females (95% CI: 1.051–1.39). In other words, male smokers smoke 19.6% more number of cigarettes than female smokers. Second, age was positively related to smoking intensity, with a 5% increase in the number of cigarettes smoked for every 10-year increase in age (95% CI: 1.008–1.001).

Marital status was also found to be a significant predictor of smoking intensity. The number of cigarettes smoked per day by divorced/widowed adult smokers was 16.8% higher than single smokers (95% CI: 1.015–1.39).

Educational attainment was another important predictor, with smokers who had a master’s degree or higher smoking 32.5% fewer the number of cigarettes than their illiterate counterparts (95% CI: 0.492–0.926).

Employment status was also found to be a significant predictor, smokers who were students (95% CI 0.522–0.917), housewives (95% CI 0.606–0.895), or unemployed with income (95% CI 0.697–0.923) smoked 31.7%, 25.8%, and 19.6% fewer the number of cigarettes than those who were unemployed, respectively.

A history of cardiovascular disease in male relatives (father, brother, or son) was found to be a protective factor for smoking intensity, with adult smokers with a history of cardiovascular disease in their male relatives smoked 15% fewer cigarettes than those who did not have such a history (95% CI 0.771–0.951).

Similarly, smoking prohibition regulations at work or school were found to be associated with lower smoking intensity, with smokers who were subject to such regulations smoking 2% fewer the number of cigarettes than those who were not (95% CI 0.843–0.942).

Finally, the number of smoking days per week was also a significant predictor of smoking intensity. Specifically, occasional smokers (i.e., those who smoked less than one day per week) smoked approximately 13 times more cigarettes than non-smokers, while smokers who smoked almost every day of the week smoked approximately 45 times more cigarettes than non-smokers.

## Discussion

The intensity of smoking is a significant factor in causing many serious smoking-related diseases, such as cancer and cardiovascular diseases. In the current longitudinal study, we investigated the factors affecting the intensity of smoking among adult smokers in the urban population of Iran. Our findings indicate that demographic factors (such as age, sex, marital status, education level and employment status), implementation of smoking prohibition regulations at work or school, and history of premature cardiovascular disease in male relatives are associated with smoking intensity among adult smokers.

Previous studies on the Iranian population have reported different trends in smoking prevalence. Nemati et al. reported stable smoking rates among Iranian men and women from 2006 to 2009 [20], while Fahimfar et al. found a decrease in smoking prevalence in both genders between 2007 and 2016 in their STEPS-based study [21]. Following Iran’s accession to the World Health Organization Framework Convention on Tobacco Control (FCTC), several laws and strategies were implemented to control tobacco consumption [22]. However, the 75% increase in cigarette prices through a WHO-recommended tax has been poorly implemented in most low- and middle-income countries, including Iran, leaving significant room for taxation as an effective and efficient tool for implementing health policies [23–25]. Rai et al. reported that implementing WHO policies on tobacco taxation could reduce the number of smokers by more than half a million [23]. Despite the effectiveness of population-based government programs, such as education on the dangers of tobacco and cigarette advertising restrictions, evidence suggests that stricter rules and more precise implementation strategies are needed to achieve long-term goals.

Numerous studies have been conducted to identify the risk factors associated with adult smoking in Iran and around the world [26–30]. However, limited research has focused on investigating the correlates of smoking intensity, which underscores the importance of considering the number of cigarettes smoked per day as a count response variable and exploring the factors related to it [31–34]. To the best of our knowledge, no study has investigated the factors associated with smoking intensity in the Iranian adult population while accounting for zero-inflation and overdispersion in this count response variable.

Male smokers smoked more number of cigarettes than female smokers. This finding is consistent with a study on Iranian adolescents and a study on young people in Turkey [34, 35]. It also complements previous research that reported a positive association between gender and smoking [28, 30, 36]. This result highlights the important role of male gender in increasing the intensity of smoking among adult smokers in Iran. Of course, it should also be noted that the habit of smoking in Iran is different compared to other regions of the world, so that smoking in Iran is not an accepted behavior among women. However, it should be noted that smoking in Iran is not an accepted behavior among women, resulting in a lower rate of smoking among women. Nevertheless, due to social prohibition, women’s self-reporting regarding smoking may not be reliable [37].

The present study also found that smoking intensity increases with age among adult smokers, which is similar to studies done in Ghana [38] and among Ethiopian men [31]. This result complements the findings of a national research in Iran, where a statistically significant difference was observed in terms of consumption in age groups, and with increasing age, the amount of smoking increased [36]. The observed age-related increase in smoking intensity may be attributed to the greater tobacco use experience and longer smoking history among older individuals.

Additionally, the study found that divorced and widowed adult smokers smoke more cigarettes per day than single individuals. This result is different from a study done in Ethiopia [31], but complements the results of a national study in Iran [27], as well as studies conducted in Kenya [39], and Nepal [40], which reported that formerly married individuals were more likely to smoke compared to their unmarried counterparts. Moreover, the study found no significant difference in smoking intensity between married and single adult smokers. These findings contradict a study that reported Iranian married individuals smoke more than single individuals [41]. However, based on the present study’s findings, the intensity of smoking is the same between married and single adult smokers.

Our study found that smokers with higher levels of education tend to smoke fewer cigarettes per day than those who are illiterate. This result is consistent with studies conducted in China [42], Ghana [38], and Nepal [40]. and complements previous findings that show a lower smoking prevalence in individuals with master’s degrees or higher, and a moderate decrease in smoking with increasing education levels [27, 43]. While some studies have shown that educated individuals smoke less overall [41, 44, 45]; our examination specifically revealed that adult smokers with master’s degrees or higher smoke fewer cigarettes per day than their illiterate counterparts. It is worth noting that our finding differs from two studies done in Ethiopia [31, 32]. Overall, our results suggest that higher levels of education in society are associated with increased awareness of the complications and diseases related to smoking, as well as the economic burden it imposes, which may lead to decreased smoking or smoking cessation.

Furthermore, the study found that student, housewife and unemployed with income smokers smoke fewer cigarettes per day than unemployed counterparts. This finding is different from a study done in China, where the intensity of smoking in employed individual (self-employed and employed by others) was lower than unemployed individual [42], but can complement the finding a national study, where odds unemployed individuals were more to smoke than housewife [27]. These results underscore the significant impact of unemployment on smoking intensity among adults and emphasize the importance of paying attention to the social and health harms caused by unemployment, such as smoking. However, the study found no significant difference in smoking intensity between employed and unemployed adult smokers.

The findings of this study suggest that a history of premature cardiovascular disease in male relatives (such as father, brother, or son) may serve as a protective factor against smoking intensity in adult males. Specifically, adult smokers with a family history of cardiovascular disease smoke fewer cigarettes per day compared to those without such a history. This finding may indicate that individuals with a family history of cardiovascular disease are more aware of the risks associated with smoking and are more likely to take steps to prevent smoking or reduce their consumption. This study contributes to the increasing body of evidence indicating that family history may have a significant impact on smoking behavior.

The present study found that adult smokers who are subject to smoking prohibition regulations at work or school tend to smoke fewer cigarettes than those without such regulations. This finding is consistent with previous studies that have reported a general reduction in smoking at the community level following the implementation of smoking prohibition in public places [46, 47]. In conclusion, this study finding complement earlier research and emphasize the critical importance of strictly enforcing smoking prohibition regulations in a range of settings, including government organizations, offices, public places, and universities. These results suggest that smoking prohibition regulations can be an effective strategy for reducing smoking behavior.

Overall, these findings suggest that smoking prohibition regulations can be an effective strategy for reducing smoking behavior. Future research should continue to investigate the mechanisms underlying this relationship, as well as explore how best to implement and enforce such regulations to maximize their impact. By better understanding the factors that influence smoking behavior and developing more effective interventions, we can work towards reducing the burden of smoking-related diseases and improving public health outcomes.

Cigarettes are a common form of tobacco and a significant risk factor for public health. Previous studies have shown that unhealthy childhood experiences, such as parental alcohol and tobacco use, can increase the risk of tobacco use in adulthood [48]. Additionally, since adults are often role models for teenagers, heavy smoking among adults may contribute to incorrect beliefs about smoking among youth. Therefore, it is essential to identify factors related to smoking intensity among adult smokers.

In a study conducted by Mohamadnejad et al., demographic factors were found to have a greater impact on smoking intensity than economic factors [41]. Thus, the present study focused on identifying demographic factors associated with smoking intensity. This research is one of the few studies of its kind in Iran, as it investigated the factors related to smoking intensity (measured by the number of cigarettes smoked per day) among adult smokers based on the Tehran Lipid and Glucose Study. The study used a mixed effects zero-inflated discrete Weibull (ZIDW) regression model with a Bayesian approach, which is more suitable for longitudinal count data that are zero-inflated and skewed (contains outliers).

One of the advantages of using the Tehran Lipid and Glucose Study data is that the sample selection process was representative of the urban society of Tehran, making the study results generalizable to the target population [49]. Additionally, the study adjusted for the variable effect of the number of smoking days per week, which is important since occasional smokers smoke fewer days per week than daily smokers.

However, the study has some limitations. First, data collection relied on self-reporting, which increases the possibility of error due to false reports. Second, the participants of the TLGS were limited to the urban areas of Tehran, so the results may not be generalizable to suburban or rural areas. Nevertheless, this limitation may be negligible since 70% of Iran’s population lives in urban areas.

## Conclusion

In conclusion, our study suggests that demographic factors are associated with the intensity of smoking among adult smokers. Therefore, it is important for policymakers to consider this population when planning interventions to reduce and prevent smoking. Furthermore, these findings highlight the need for continued longitudinal research to identify additional factors that may contribute to changes in smoking intensity over time.

## Data Availability

The datasets used and/or analyzed during the current study are available from the FA, principal investigator (PI) of the TLGS on reasonable request. *azizi@endocrine.ac.ir*.

## Abbreviations

TLGS: Tehran Lipid and Glucose Study
ZIDW: Zero inflated discrete Weibull
JAGS: Just Another Gibbs Sampler
PE: Posterior estimate
HPD: Highest posterior density
